# Exploring how paramedics are deployed in general practice and the perceived benefits and drawbacks: a mixed-methods scoping study

**DOI:** 10.3399/bjgpopen20X101037

**Published:** 2020-05-13

**Authors:** Behnaz Schofield, Sarah Voss, Alyesha Proctor, Jonathan Benger, David Coates, Kim Kirby, Sarah Purdy, Matthew Booker

**Affiliations:** 1 University of the West of England UWE Bristol, Bristol, UK; 2 University Hospitals Bristol NHS Foundation Trust, Bristol, UK; 3 BrisDoc Healthcare Services, Bristol, UK; 4 South Western Ambulance Service NHS Foundation Trust, Exeter, UK; 5 Bristol Medical School, University of Bristol, Bristol, UK

**Keywords:** paramedic, allied health personnel, primary health care, general practitioners, health workforce

## Abstract

**Background:**

General practice in the UK faces continuing challenges to balance a workforce shortage against rising demand. The NHS England *GP* *Forward View* proposes development of the multidisciplinary, integrated primary care workforce to support frontline service delivery, including the employment of paramedics. However, very little is known about the safety, clinical effectiveness, or cost-effectiveness of paramedics working in general practice. Research is needed to understand the potential benefits and drawbacks of this model of workforce organisation.

**Aim:**

To understand how paramedics are deployed in general practice, and to investigate the theories and drivers that underpin this service development.

**Design & setting:**

A mixed-methods study using a literature review, national survey, and qualitative interviews.

**Method:**

A three-phase study was undertaken that consisted of: a literature review and survey; meetings with key informants (KIs); and direct enquiry with relevant staff stakeholders (SHs).

**Results:**

There is very little evidence on the safety and cost-effectiveness of paramedics working in general practice and significant variation in the ways that paramedics are deployed, particularly in terms of the patients seen and conditions treated. Nonetheless, there is a largely positive view of this development and a perceived reduction in GP workload. However, some concerns centre on the time needed from GPs to train and supervise paramedic staff.

**Conclusion:**

The contribution of paramedics in general practice has not been fully evaluated. There is a need for research that takes account of the substantial variation between service models to fully understand the benefits and consequences for patients, the workforce, and the NHS.

## How this fits in

Despite the clear policy direction, very little is known about the safety, clinical effectiveness, and cost-effectiveness of paramedics working in general practice. Furthermore, there is limited information available about the different ways paramedics are deployed in general practice, and the intended benefits and unintended consequences of different workforce models. This scoping study explores the theories underpinning the deployment of paramedics in general practice services. The findings can be used to inform larger scale research on this subject.

## Introduction

General practice services in the UK are facing an unprecedented recruitment and retention challenge. This is happening at a time when services are under increasing pressure owing to a growing and ageing population.^1,2^ The NHS England *GP* *Forward View* seeks to address workload issues in primary care by promoting a multidisciplinary approach. It proposes funding for 20 000 more staff to be enlisted to support GPs. These staff, including paramedics, are intended to free up GPs to spend more time with patients and enable practices to offer more services.^3^ Some of the perceived benefits of deploying paramedics in general practice are highlighted as the management of minor illnesses, undertaking home visits, and the provision of same-day ‘urgent’ primary care.^4,5^

Precise figures on the number of paramedics working in general practice are difficult to ascertain owing to the wide variety of employment models. However, the General Practice Workforce dataset indicates that numbers almost doubled between 2017 and 2018.^6^ Despite this, and the clear policy directive, very little is known about the safety, clinical-, or cost-effectiveness of paramedics working in general practice. Much of the existing literature focuses on which additional skills may be needed by paramedics to work autonomously and safely in general practice and other community settings.^7–9^ However, this research is largely descriptive and there are several assumptions, such as paramedics reducing GP workload and costs, which have not been tested empirically.

A particular challenge for general practice is embedding paramedics in the most appropriate way. Local need will, to some extent, dictate the types of patients seen, the clinical problems managed, and the relationships with other acute and community services.^10^ Different practice sizes, workforce composition, and demographics mean it is likely that a range of models are already in place. For example, paramedics may be employed directly by a practice or shared across a primary care network or clinical commissioning group (CCG). Some paramedics may be employed for specific tasks, while others may work more broadly in general practice. Before research can begin to determine the most appropriate ways of utilising paramedics in the general practice workforce, it is important to understand the extent of variation and associated drivers.

The aim of this scoping study is to explore the current provision of paramedics in general practice, and describe what the intended benefits and unintended consequences of this workforce organisation might be.

The objectives of this study are to:

- identify existing evidence on the effects of deploying paramedics in general practice;
- characterise different models for deploying paramedics; and,
- understand the hypotheses that underpin the models and explore the potential for unintended consequences for patients, staff, and the wider NHS.

## Method

A mixed-methods scoping study was undertaken in three phases.

### Phase 1: Literature review and survey

In order to map the existing deployment of paramedics in general practice, a systematically-searched scoping review of the literature including key national documents on the topic was completed. A web-based survey of paramedics, and staff working with them, in general practice was also carried out.

#### Literature review

Databases and search terms were agreed by the research team with the support of a subject specialist librarian (Table 1). Relevant articles and documents were identified by title and abstract, and a second reviewer repeated this process.

**Table 1.** Literature review search strategy and summarised results

**Search strategy and terms**
’GP*‘ OR ’general practitioner*‘ OR ’family practitioner*‘ OR ’family physician*‘ OR ’family doctor*‘ OR ’primary care‘ OR ’primary healthcare‘ OR ’primary health care‘ OR ’primary practice*‘ OR ’general practice*‘ OR ’family practice*’AND ‘paramedic*’ OR ’emergency care practitioner*‘ OR ’urgent care practitioner*’Limited to English.Databases: Medline, Cochrane, EMBASE, Psych INFO, AMED, CINHAL plus				
**Records identified**				
Records excluded at title and abstract screening, *n* = 3017Full texts for screening, *n* = 29Full texts for screening after duplicates removed, *n* = 17 (12 duplicates found)Full texts screened for relevance after reading articles, *n* = 14				
**Literature review summary (*n* = 14**)				
**Year**	**Author**	**Title**	**Publication**	**Summary**
2005	Ball L^11^	Setting the scene for the paramedic in primary care: a review of the literature	*Emergency Medicine Journal*	Explores the published evidence that surrounds paramedic practice in an attempt to identify the skills, training, and professional capacity that paramedics of the future will require. Identified the paucity of published evidence. Concluded that paramedics must work together to take ownership of the basic philosophies of their practice, which must have their foundation in valid and reliable research.
2012	Daly J^12^	The paramedic in the community: my story	*Primary Health Care*	Aims to provide a clear picture of the role of paramedics based in the community and show how this role has developed through the pioneering personal experiences of a paramedic working in a medical practice. It highlights, through the responses to a questionnaire, how paramedics could be an effective additional resource to an established primary care team.
2018	Eaton G,Mahtani K,Catterall M^13^	The evolving role of paramedics — a NICE problem to have?	*Journal of Health Services Research and Policy*	Supports the growing role of paramedics in the clinical and academic workforce. A commentary on recent draft consultations by the National Institute for Health and Care Excellence (NICE) in England that set out how the role of paramedics may be evolving to assist with the changing demands on the clinical workforce and suggest that the profession should also lead the academically driven evaluation of these new roles.
2014	Evans R,McGovern R,Birch J,Newbury-Birch D^14^	Which extended paramedic skills are making an impact in emergency care and can be related to the UK paramedic system? A systematic review of the literature	*Emergency Medicine Journal*	A total of 8724 articles were identified, of which 19 met the inclusion criteria. Fourteen articles considered paramedic patient assessment and management skills, two articles considered paramedic safeguarding skills, two health education and learning sharing, and one health information. There is valuable evidence for paramedics assessing and managing patients autonomously to reduce emergency department conveyance, which is acceptable to patients and carers. Evidence for other paramedic skills is less robust, reflecting a difficulty with rigorous research in pre-hospital emergency care.
2018	Mahtani KR,Eaton G,Catterall M, Ridley A^4^	Setting the scene for paramedics in general practice: what can we expect	*Journal of the Royal Society of Medicine*	Current opportunities for employment in NHS general practices still require careful evaluation for context-specific clinical outcomes, value, and satisfaction.
1974	Marsh GN,McNay RA^15^	Team work load in an English general practice	*British Medical Journal*	A survey of a general practice demonstrated that by delegating work to a team of paramedical workers, by increasing the proportion of personal medicine, and by engaging the cooperation of patients the GP reduced his workload considerably without any apparent reduction in standard of care.
2009	Martin-Misener R,Downe-Wamboldt B,Cain E, Girouard M^16^	Cost effectiveness and outcomes of a nurse practitioner-paramedic-family physician model of care: the Long and Brier Islands study	*Primary Health Care Research and Development*	The study in a rural Canadian setting evaluated nurse practitioners, paramedics, and family physicians providing care together. This model of care resulted in decreased cost, increased access, and a high level of acceptance and satisfaction among care providers.
2018	Moule P *et al* ^7^	Preparing non-medical clinicians to deliver GP out-of-hours services: lessons learned from an innovative approach	*Education for Primary Care*	This article presents the development and evaluation of one programme delivered in 2017 to paramedics seeking to work in out-of-hours (OOHs) services. The findings suggest the course was able to provide the students with the skills, knowledge, and confidence to become safe practitioners in the OOHs service.
2019	Rasku T *et al* ^9^	The core components of community paramedicine — integrated care in primary care setting: a scoping review	*Scandinavian Journal of Caring Sciences*	The Community Paramedicine programmes are perceived to be promising. However, Community Paramedicine research data are lacking. Further research is required to understand whether this novel model of health care is reducing costs, improving health, and enhancing people's experiences.
1983	SaintYves IF^17^	The training of paramedics for primary health care	*Journal of the Royal Society of Health*	Defends the use of appropriately trained paramedics as first point of contact in the primary care setting to alleviate staff shortages.
2017	Spence D^5^	Good medicine — the GP paramedic	*British Journal of General Practice*	A one-page personal commentary from a GP in Glasgow supporting the role of paramedics in GP settings and proposing the name changes to GP doctor, GP nurse, and GP paramedic to allow for the changes to the healthcare team.
1974	Willemain TR,Moore GT^18^	Planning a medical practice using paramedical personnel	*Health Services Research*	Using a mathematical model the paper sets out a planning methodology to decide on the trade-offs between key parameters in terms of type of paramedic, size of practice, costs, and so on to help determine the impact of the use of paramedics in primary care.
2006	Woollard M^8^	The role of the paramedic practitioner in the UK	*Journal of Emergency Primary Health Care*	Describes the evolution of the role of the paramedic practitioner in the community and the formalisation of the training needs for paramedics to work autonomously in the community setting.
2016	Schadewaldt V,McInnes E, Hiller J, Gardner E^19^	Experiences of nurse practitioners and medical practitioners working in collaborative practice models in primary healthcare in Australia — a multiple case study using mixed methods	*BMC Family Practice*	This study investigated the experiences and perceptions of nurse practitioners and medical practitioners who worked together in Australia and found that the willingness of practitioners and their individual relationships partially overcame the effect of system restrictions. However, this article highlights the need for strategic support to ensure the sustainability of collaborative practice models in primary health care.

#### Survey

The survey contained 10 items and requested a mixture of categorical and free-text responses. It was developed in Qualtrics (version XM) by the study team in consultation with a wider stakeholder group and piloted with paramedics, GPs, practice managers, and commissioners. It was then administered to a convenience sample during June and July 2019 by the distribution of a web link as an open survey. The survey was cascaded by liaising with all local medical committees in England and distributed through a variety of CCGs, including national communication, and by utilising paramedic networks and social media. Consent to participate was obtained. There was no incentive offered for completion. Responses were anonymised and no identifiable data were collected.

### Phase 2: Key informant (KI) meetings

Key informants (KIs) were met from two CCGs in the south west of England and professional bodies to identify existing theories about the intended outcomes of deploying paramedics in general practice. These meetings were also used to identify potential participants for phase 3. KIs were individuals employed within directly relevant organisations who had specific knowledge about, or experience of, paramedics working in general practice. KIs were identified by the study team using existing contacts and a snowball approach. The meetings between the researcher and each KI took place during April and May 2019 at a mutually agreed time and location. KIs gave written informed consent. Meetings lasted 30–40 minutes and were audiorecorded to supplement note-taking.

### Phase 3: Stakeholder (SH) interviews

Direct enquiry with stakeholders (SHs)was used to examine the underlying assumptions about how different approaches to paramedic deployment were intended to work. A topic guide was developed by the research team in advance of the interviews and used to guide the interviews. The SHs were from two local CCGs in the south west of England. Participants were identified during phase 2 and were eligible if they were staff working in general practices with paramedics. The SHs were approached by email, and semi-structured face-to-face interviews were conducted by a researcher at a mutually agreed time and location. Participants gave written informed consent. Interviews lasted 30–50 minutes and were audiorecorded with an encrypted device, anonymised, and transcribed verbatim. Transcripts were analysed thematically by the primary researcher to examine the underlying assumptions about how different approaches to paramedic deployment are intended to work.^20^ Codes and categories were developed by the study team and 20% of the transcripts were double-coded by a second researcher.

## Results

### Phase 1

#### Literature review

Fourteen articles dating back to 1974 were included in the review (Table 1). The majority of articles are commentaries on the evolving role of paramedics and the training and skills required for paramedics to work in primary care settings. Recent commentaries support the role of paramedics in primary care settings and recognise the need to develop this further.^2,4,5,8,11–13^ However, there is no empirical evidence on the safety, clinical-, or cost-effectiveness of paramedics working in general practice. Searches of the grey literature returned descriptive documents, including job specifications, experiences of local initiatives, and discussions of paramedics’ skills. No key national documents were identified.

#### Survey

A total of 165 responses were received in a 4-week period. Eighty-seven (52.7%) responders specified the CCG in which they worked. Forty-five (23.6%) of 191 CCGs were specified; covering North of England, the Midlands, London, South East England, and South West England. One-third of the specifications were concentrated in South West England, with the remainder evenly spread across the other areas.

Seventy-five (45.5%) responders were paramedics, 32 (19.4%) were GPs, 40 (24.2%) were practice or business managers, and 18 (10.9%) were other staff such as nurses or pharmacists.

The majority of paramedics are employed directly by the GP practice. Others work across a primary care network, or are employed by a CCG, local ambulance service, or a community provider (Table 2). Ninety (54.5%) responders reported that paramedics had relevant post-registration qualifications. The reported qualifications were: clinical masters degree (MSc) (*n* = 17); diploma (*n* = 13); postgraduate certificate (*n* = 10); non-registered course (*n* = 18); working towards MSc or diploma (*n* = 13); did not specify, or referred to registration qualification (*n* = 19).

**Table 2.** Model of employment and tasks undertaken by paramedics

**Model of employment**	***n***	**%**
Directly employed by GP practice	98	59.4
Directly employed by CCG	2	1.2
Contract with community provider	2	1.2
Freelance or locum contract	0	0.0
Agency	0	0.0
Contract with local ambulance trust	14	8.5
Other	8	4.8
PCN	6	3.6
Combination of GP practice and PCN	10	6.1
Other combination	16	9.7
Missing	9	5.5
Total	165
**Tasks undertaken** ^a^
Same-day home visits	145	91.8
Routine home visits	97	61.4
Same-day clinic	118	74.7
Pre-booked clinic	53	33.5
Same-day telephone triage	56	35.4
Pre-booked telephone triage	21	13.3
Telephone triage	68	43.0
Web triage	8	5.1
Other tasks	22	13.9
Total	588	
**Exclusions reported** ^b^
No exclusions	27	24.1
Aged <6 months	25	22.3
Aged <1 year	23	20.5
Aged <2 years	12	10.7
Aged <5 years	5	4.5
Pregnant women	22	19.6
Sexual health or gynaecology	17	15.2
Mental health	20	17.9
End of life or palliative care	14	12.5
Chronic or complex conditions	14	12.5
Other specified condition^c^	12	10.7
Total^d^	164	

^a^Denominator 158; 7 responders did not answer/didn’t know. ^b^Denominator 112; 53 responders did not answer/didn’t know. ^c^For example, moles, lumps, patients in care homes, and decision made by paramedic. ^d^Some responders reported more than one exclusion. CCG = care commissioning group. PCN = primary care network.

The tasks paramedics are undertaking are mostly same-day home visits (91.8%), followed by same-day clinics (74.7%), routine home visits (61.4%), and telephone triage (43.0%). A third of responders also reported that paramedics do pre-booked clinics (*n* = 53; 33.5%) and same-day telephone appointments (*n* = 56; 35.4%) (Table 2).

There was significant variation in reports on the types of condition and patient groups that paramedics are employed to see. This ranged from seeing all patients to focusing on acute presentations, older patients, or housebound patients. A total of 112 (67.9%) participants responded to a question about the types of patients that would not be seen by a paramedic. Of these, 137 (76%) reported one or more patient groups as exclusions. The most common exclusions were infants, pregnant women, and patients with mental health needs (Table 2).

**Figure 1.**
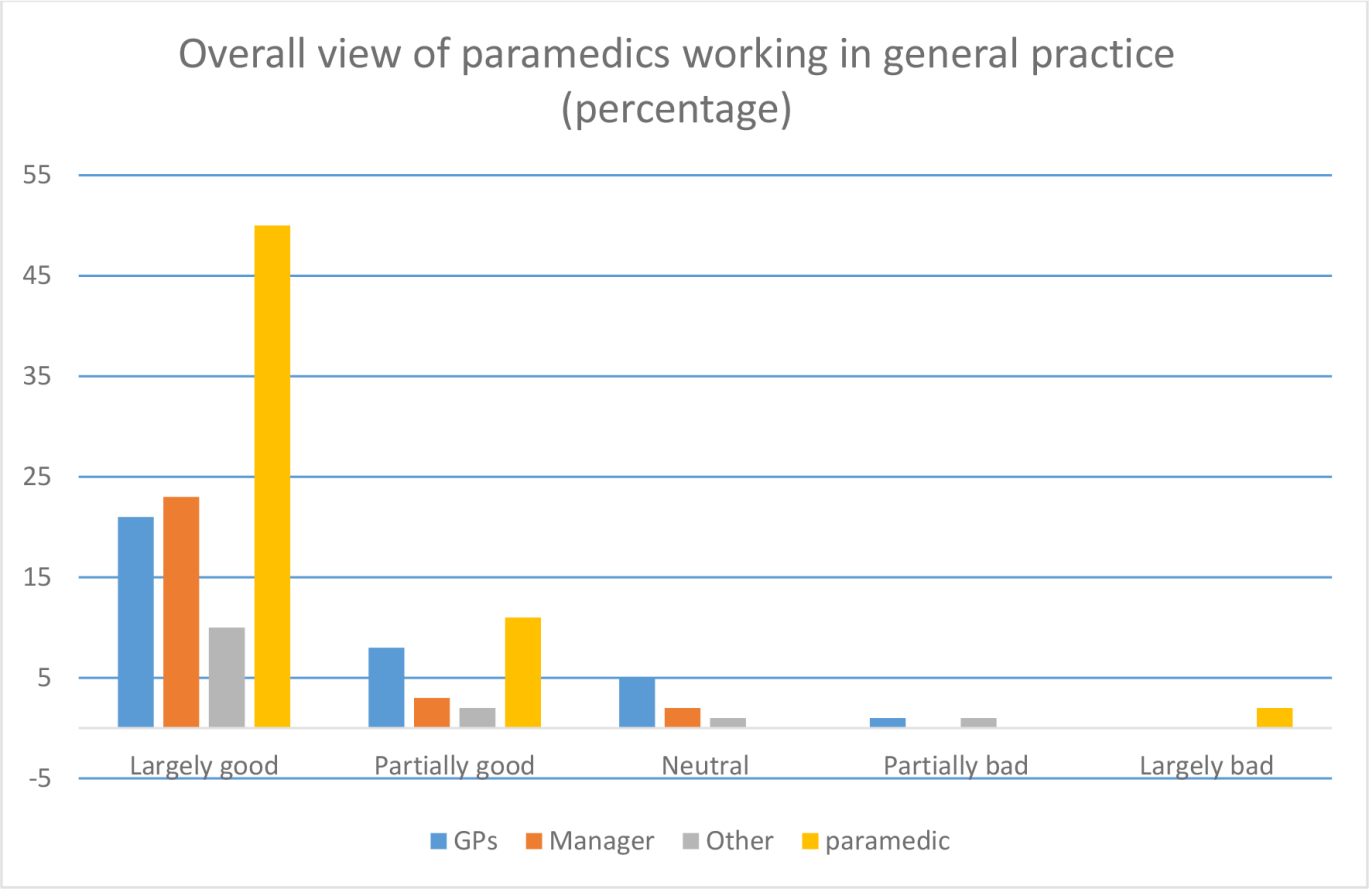
Overall view of paramedics working in general practice. Missing data *n* = 25.

The survey collected views on paramedics working in general practice. A total of 140 (84.8%) expressed a view; of these, 104 (74.3%) felt that paramedics working in general practice was a largely positive initiative (Figure 1).

### Phase 2: Key informant meetings

Nine KIs were approached and agreed to meet (Table 3). All KIs described their involvement in the planning, commissioning, or staff education of unplanned, urgent care, and primary care services within their localities. Some described roles and responsibilities specifically relating to workforce planning and development. The KIs were not aware of any CCG-led policy initiative to place or support paramedics in general practice. In their experiences, deployment of paramedics had been initiated at a practice level and evolved gradually over the previous 5 years. KIs spoke about the shortage of GPs and experienced advanced nurse practitioners, and acknowledged a need to deploy other healthcare professionals. Paramedics are recognised as clinicians with a skillset that is a valuable addition in primary care, rather than being used to replace GPs or deputise for them. No information is collected on paramedics working in GP practices by the CCG. KIs were less aware of the different nuanced models of paramedic deployment within primary care services. The advantages of paramedics working in primary care services were mostly envisaged as home visits and urgent cases being seen in a timelier manner.

**Table 3.** Key informant and stakeholder characteristics

**KI meetings**
	**Sex**	**Role**	**Employer**
KI 1	Male	GP	CCG
KI 2	Female	GP	CCG
KI 3	Female	Commissioner	CCG
KI 4	Male	GP	CCG
KI 5	Male	Paramedic	CCG
KI 6	Female	Commissioner	CCG
KI 7	Male	Paramedic	CCG
KI 8	Female	Paramedic	Professional body
KI 9	Female	Paramedic	NHS
**SH interviews**			
SH 1	Female	Paramedic	
SH 2	Female	GP	
SH 3	Female	Practice manager	
SH 4	Female	GP	
SH 5	Male	GP	
SH 6	Male	GP	
SH 7	Male	Practice manager	
SH 8	Female	Practice manager	
SH 9	Female	Paramedic	
SH 10	Female	GP	

CCG = clinical commissioning group. KI = key informant. SH = stakeholder.

KIs expressed concern that not all paramedics would be well-placed in general practice, depending on career stage and experience. However, they also identified the possibility that if more educated and experienced paramedics were to leave the ambulance service, the less experienced staff remaining could result in an increased conveyance rate to hospital. However, some KIs felt that if these paramedics were going to leave the ambulance service, employment in general practice is a way of retaining their skills within the NHS. There is a sense that paramedics are interested in portfolio careers and that the ambulance service is no longer seen as a 'job for life'. The rotational model, which is being piloted in England, was mentioned by some of the KIs as a model that could be replicated more widely.^21^

### Phase 3: Stakeholder interviews

Ten SHs were approached and all agreed to be interviewed (Table 3). Codes were discussed by the study team and three themes emerged: the future of primary care; benefits and consequences for primary care; and professional challenges.

#### The future of primary care

The landscape of general practice is changing. There are not enough GPs; therefore, adaptive staff recruitment is required to meet the needs of patients. Although increasing GP numbers is seen as a priority, some clinical tasks can be undertaken by paramedics, which may save money:

*'A very expensive, highly trained person doing every type of clinical role across the practice doesn’t make sense when you could employ people … who might be cheaper.'* (SH6)

Conversely, this may not necessarily be seen as an effective use of resources:

*'* *When I look at the home visits … we send the paramedic and then we send the paramedic the second time and then we send the GP the third time. So have we done more than we would've done normally and* *…* *I'm not convinced that we've actually saved anything.* *'* (SH3)

Continuity of care is a central tenet of general practice.^22^ In some cases, paramedics who undertake home visits can support this:

*'*[Paramedic] *has been there for four years* *…* [patients] *get to know and trust the practitioners and they’re not just paramedics.* *'* (SH4)

#### Benefits and consequences for primary care

In general, paramedics are seen as a positive addition to the general practice team; reducing demand on GP time and ensuring more patients are seen sooner:

*'* *So I think it benefits us but it also really benefits our patients because …* [the paramedic] *can say “Look I’ll be with you, you know, in half an hour” and we didn’t have that before.* *'* (SH2)

*'* *For those patients where actually it's more of a social need to visit, then* [the paramedic] *are absolutely the right person who can spend half an hour where a GP can't.* *'* (SH3)

However, there are resource implications for the rest of the practice and other staff:

*'* *GPs will say ”Well hang on, you know, I don’t want them spending forty, fifty minutes in the house* *…* *I want to see that level of value for money”* *…* *'* (SH5)

The tendency for paramedics to see low-complexity cases can result in GPs primarily seeing patients with multimorbidity, chronic illness, and, often, social complexities. This has the potential to impact on the job satisfaction and mental wellbeing of GPs:

*'* *But if the paramedics see all of these* *“* *easy stuff* *”* *…* *you're left with only the really complicated stuff* *…* *I found that emotionally very hard work and draining.* *'* (SH4)

This may to some extent be explained by differences in clinical practice, as described by this paramedic:

*'* *I guess that I'm able to give them a bit more time than a busy GP can and with that time I can use it to maybe do a fuller assessment* *…* *I have less knowledge so I need to check more things to make sure I'm not missing anything* *.'* (SH1)

Nevertheless, if paramedics free up time, GPs can then complete other tasks more effectively, which reduces stress levels:

*'* *If a GP has enough time, then what feels very stressful becomes more manageable. It is a huge psychological relief* *…* *'* (SH6)

#### Professional challenges

Paramedics in general practice are generally viewed in a positive light by participants in this study; however, the success of various models depends to some extent on how they are implemented and on the individuals involved:

*'* *Perhaps all paramedics wouldn't be the same character you know, her character really she fitted in with our team really well and we’re very lucky.* *'* (SH2)

Some paramedics feel ‘out of their depth’ in general practice and do not stay in their primary care role for as long as planned:

*'* *…* *good paramedic but his skills didn’t seem to transfer into* *general practice,* *so after about six months he decided that perhaps he's better off going back as a paramedic.* *'* (SH8)

There is limited agreement about the scope of practice for paramedics. One view is that ambulance service paramedics are best trained to deal with acute presentations:

*'* *They're not very good at chronic disease management and that's essentially ‘cos they're paramedics … their job within the ambulance service is not the management of long-term diabetes or long-term COPD* [chronic obstructive pulmonary disease]*.* *'* (SH5)

An alternative view is that paramedics are well-equipped to deal with anything:

*'* *We’re trained to see from the day you’re born, from the second you’re born to the second you die and we see everything in between that … different illnesses, chronic illnesses.* *'* (SH9)

Either way, effective supervision and support are essential:

*'* *Some of the other paramedics have been forced into practice with no preparation and given timetabled slots with very little supervision and they're the ones that have found it difficult …* *'* (SH4)

However, a consequence of good support and supervision is the impact on GP time:

*'* *We can have someone that we hope is going to take some load off us and for the first six months they may actually be a liability rather than a true asset and that's a challenge.* *'* (SH6)

## Discussion

### Summary

This scoping study aimed to explore the current provision of paramedics in general practice and describe what the intended benefits and unintended consequences of this workforce organisation might be. There is significant variation in the types of models adopted and disparity over a number of issues. The majority of models reported include home visits as a key feature. However, the type of patients seen and conditions treated vary significantly. On one hand, a perceived strength of a paramedic is that they have been trained to see all patients; on the other hand, paramedics are often excluded from seeing specific patient groups. An additional argument in support of deploying paramedics is that they will free up GP time; however, in some cases the amount of training, supervision, and support that is required may initially negate this. A third component to the debate is that paramedics cost less to employ; however, they may need substantially more time than GPs to assess and treat patients, and this assumption has not been tested empirically.

### Strengths and limitations

These findings provide new information about the current provision of paramedics in general practice and what the intended benefits and unintended consequences of this workforce organisation might be. However, there are a number of limitations; the survey used a convenience sample and it is not possible to determine the response rate or whether the views expressed were representative of a wider group. The interviews were conducted within two neighbouring CCGs and this may limit the generalisability of the findings.

### Comparison with existing literature

To the authors’ knowledge, there is no published evidence regarding the safety, clinical-, or cost-effectiveness of paramedics working in general practice.^10^ This study supports previous research asserting the need to understand and monitor the broader implications of changes in the general practice workforce,^1,23,24^ and offers some theories applicable to paramedics specifically.

### Implications for research

The scale and significance of the issues discussed almost certainly varies according to local need, and further investigation is required to understand what works, and how, under differing circumstances. Research is needed to determine the effect of paramedics working in general practice on patient safety and experience, and to inform local and national decision making.
